# Propensity-score matched outcomes of minimally invasive and open pelvic exenteration in locally advanced rectal cancer

**DOI:** 10.1007/s13304-025-02102-7

**Published:** 2025-01-16

**Authors:** Sameh Hany Emile, Nir Horesh, Zoe Garoufalia, Rachel Gefen, Justin Dourado, Ebram Salama, Steven D. Wexner

**Affiliations:** 1 Ellen Leifer Shulman and Steven Shulman Digestive Disease Center, Cleveland Clinic Florida, 2950 Cleveland Clinic Blvd., Weston, FL 33179 USA; 2 Colorectal Surgery Unit, General Surgery Department, Mansoura University Hospitals, Mansoura, Egypt; 3 Department of Surgery and Transplantations, Sheba Medical Center, Ramat Gan, Affiliated with the Faculty of Medicine, Tel Aviv University, Tel Aviv, Israel; 4 Department of General Surgery, Hadassah Medical Organization and Faculty of Medicine, Hebrew University of Jerusalem, Jerusalem, Israel

**Keywords:** Minimally invasive, Open, Pelvic exenteration, Locally advanced rectal cancer, Propensity Score matched, NCDB

## Abstract

Pelvic exenteration (PE) entails an en bloc resection of locally advanced primary or recurrent rectal cancer. This study aimed to assess the short-term and survival outcomes of minimally invasive (MI)- and open PE. A retrospective cohort analysis of patients with stage III rectal adenocarcinoma treated with PE from the National Cancer Database (2010–2019) was conducted. Open and MI-PE were matched for baseline and treatment characteristics using 2:1 propensity score matching. Primary outcomes were 30- and 90-day mortality; secondary outcomes included 30-day readmission, hospital stay, surgical margins, lymph node yield, and overall survival (OS). PE was performed in 1010 (1.9%) of 52,242 patients; 705 (69.8%) were open and 304 were (30.2%) MI procedures. After matching, 169 patients in the MI-PE group were matched to 338 patients in the open PE group. The matched cohort included 507 patients (54.2% female). Hospital stay was shorter after MI compared to open PE (median: 6 vs. 8 days, *p* < 0.001). MI and open PE had similar odds of 30- (OR: 0.33, *p* = 0.306) and 90-day mortality (OR: 0.29, *p* = 0.113), and comparable rates of positive surgical margins (12.7% vs. 15%, *p* = 0.586) and suboptimal lymph node yield (19% vs. 26%, *p* = 0.096). MI-PE had a similar OS to open surgery (82.5 vs. 77.5 months, *p* = 0.281). Robotic-assisted PE was associated with significantly lower odds of conversion to open surgery (OR: 0.15, *p* = 0.003) and shorter hospital stay (median: 5 vs. 7 days, *p* = 0.026) than laparoscopic PE. MI-PE provided similar pathologic and survival outcomes to open PE with comparable short-term mortality rates and significantly shorter hospital stays.

**Supplementary Information:**

The online version contains supplementary material available at 10.1007/s13304-025-02102-7.

## Introduction

Locally advanced rectal cancer (LARC) involves tumor infiltration beyond the muscle layers of the rectum and/or regional lymph node involvement. The treatment of primary LARC includes neoadjuvant chemoradiation therapy (NCRT) followed by total mesorectal excision [1]. Surgical techniques for LARC vary according to the level and extent of rectal cancer [2]. Pelvic exenteration (PE) entails an en bloc resection of locally advanced or recurrent rectal cancer. PE typically involves multi-visceral resection of pelvic structures and can be classified into different types. Anterior PE involves the resection of gynecologic and urologic structures, posterior PE involves the resection of the colon/rectum together with gynecologic structures while preserving the urinary bladder and urethra, total PE combines anterior and posterior PE, and extended PE involves an abdominosacral resection [3]. Currently, the concept of PE expands to include bony structures, such as the sacrum and pubic bone, when radical resection is needed [4].

The main goal of PE is to achieve a clear resection margin with an R0 status [5]. Given the extensive resections involved, PE is associated with considerable rates of post-operative complications and mortality. A systematic review reported PE to be a lengthy operation with a median operative time of 7.2 h and complication rates ranging from 31.6 to 86%. Despite the extensive resections performed, the oncologic outcomes after PE are usually dismal with a median 5-year survival of 32% [6]. Minimally invasive PE was devised to decrease short-term complications and improve immediate recovery. A cohort study confirmed the safety and feasibility of minimally invasive posterior and total PE in select patients with LARC. However, minimally invasive surgery did not significantly reduce the overall morbidity rate or shorten the length of hospital stays [7]. Similarly, another study reported a similar hospital stay, R0 resection rates, and 3-year survival when comparing minimally invasive and open PE [8]. A systematic review of 170 patients, 22% of whom underwent minimally invasive PE, concluded that minimally invasive PE is feasible in selective cases and associated with lower post-operative morbidity and shorter stay [9].

Given the paucity and small sample size of the studies that compared the outcomes of minimally invasive and open PE, the present study aimed to assess the short-term and survival outcomes of the two approaches in stage III rectal cancer using a large national database. The study focused on node-positive rectal cancer as complete resection with adequate lymphadenectomy in these tumors can be challenging and associated with a considerable morbidity, specifically in the setting of PE. Our study hypothesized that using MIS in PE may help reduce short-term mortality and shorten hospital stays with a potential survival benefit.

## Patients and methods

### Study design and data source

A retrospective cohort analysis of the outcomes of open and minimally invasive PE was conducted. Data were derived from the National Cancer Database (NCDB) from 2010 to 2019. The NCDB includes hospital registry data from more than 1500 hospitals that are accredited by the Commission on Cancer (CoC) in the United States. The NCDB is recognized as a joint project of the CoC of the American College of Surgeons and the American Cancer Society. The NCDB and the hospitals participating in the CoC NCDB have not verified and are not responsible for the statistical validity of the data analysis or the conclusions derived by the authors. Approval from the ethics committee and written consent were not required given that the de-identified patient data used were derived from a public database. The study was reported in compliance with the STROBE guideline and the EQUATOR guidelines for PSMA reporting (Supplementary Table 1) [10].

### Study population

Patients included in the study underwent PE for stage III rectal adenocarcinomas (International Classification of Diseases for Oncology, 3rd edition ICDO-3 code 8140/3, 8480–8481/3, 8490/3) who underwent PE. The NCDB does not differentiate between types of PE. Therefore, all types of PE were eligible for inclusion. We excluded patients with rectal cancer of other or unknown clinical stages, patients who did not have surgery, patients who had other types of surgery or non-specified surgery, and patients with an unknown surgical approach.

### Data points

Data used for the analysis comprised patient characteristics (age, sex, race, Charlson comorbidity index score, insurance status, and facility type); tumor characteristics (clinical TNM stage, histology, grade); and treatment details (neoadjuvant radiation and systemic therapy and approach of surgery).

### Study outcomes

The primary outcome of the study was 30- and 90-day mortality of open and minimally invasive PE. Secondary outcomes included 30-day readmission, hospital stay, surgical margins, examined lymph node number, and 5-year overall survival (OS). OS was calculated from the time of surgery to the date of last contact or death due to any cause. The outcomes of laparoscopic and robotic PE were compared as a secondary outcome of the study.

### Bias

We consecutively included patients from a large national database and used propensity score matching to minimize sampling and selection bias.

### Statistical analysis

EZR™ (version 1.55) and R software (version 4.1.2) were used to conduct the statistical analyses in this study. We presented normally distributed continuous data as mean and standard deviation or otherwise as median and interquartile range (IQR). Student’s t test or Mann–Whitney test was used for analysis as appropriate. Categorical data were presented as numbers and proportions, and analyzed using the Fisher’s exact or chi-square test, as appropriate.

Patients who underwent PE were divided into two groups: open and minimally invasive surgery (laparoscopic and robotic-assisted surgery). The two groups were matched using the nearest neighbor, 2:1 propensity score method with a caliper of 0.2 and without replacement. Since the number of patients who underwent open PE was more than twice those who underwent minimally invasive PE, we used a 2:1 matching to simulate the original cohort and maintain external validity [11]. A caliper of 0.2 was used to obtain a larger matched cohort and increase precision as previously suggested for propensity score matching [12].

The matching criteria included clinically relevant covariates that showed baseline imbalance with a standardized mean difference (SMD) > 1. An SMD ≤ 0.1 for all baseline factors after matching indicated the balance of the matched groups. The analysis involved the average effect of the treatment on the treated (ATT) since it aimed to assess the effect of the approach of PE on short-term outcomes. Sensitivity analyses using the Mantel–Haenszel method were conducted. Gamma values indicate the setting of the sensitivity parameter used and the lower and upper p values represent the lower and upper bound of the confidence interval for the Mantel–Haenszel statistic. Differences in OS were assessed using Kaplan–Meier statistics and the log-rank test. Missing data were addressed using a complete case analysis. The significance level was set at 5%.

## Results

### Original unmatched cohort

**Fig. 1:**
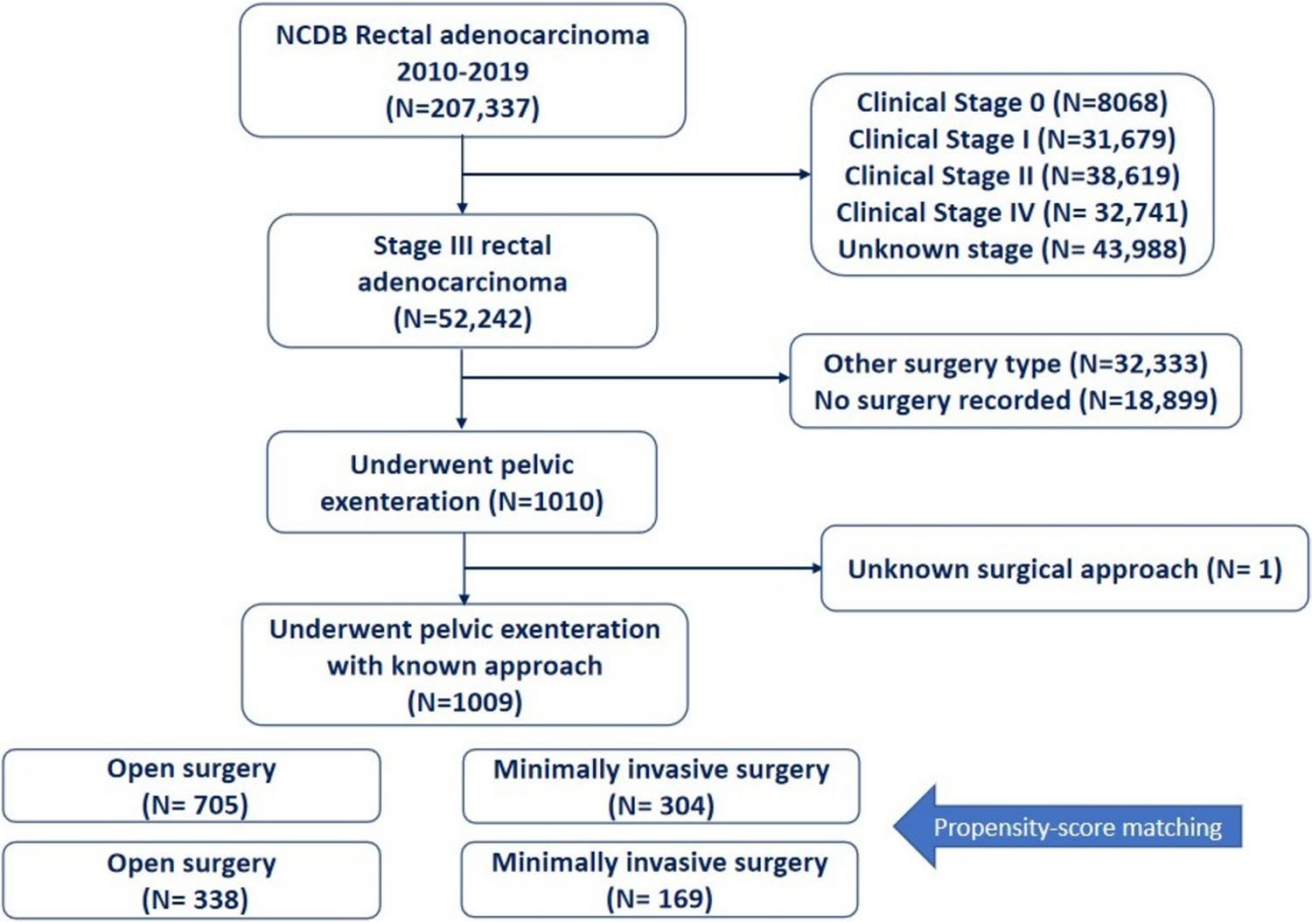
Flow chart for patient inclusion in the study

PE was performed in 1010 (1.9%) of 52,242 patients with stage III rectal adenocarcinoma. The surgical approach was not recorded in one patient; thus, 1009 patients were finally included in the study (Fig. 1). Patients were 529 (52.4%) female with a mean age of 58 years.

**Fig. 2:**
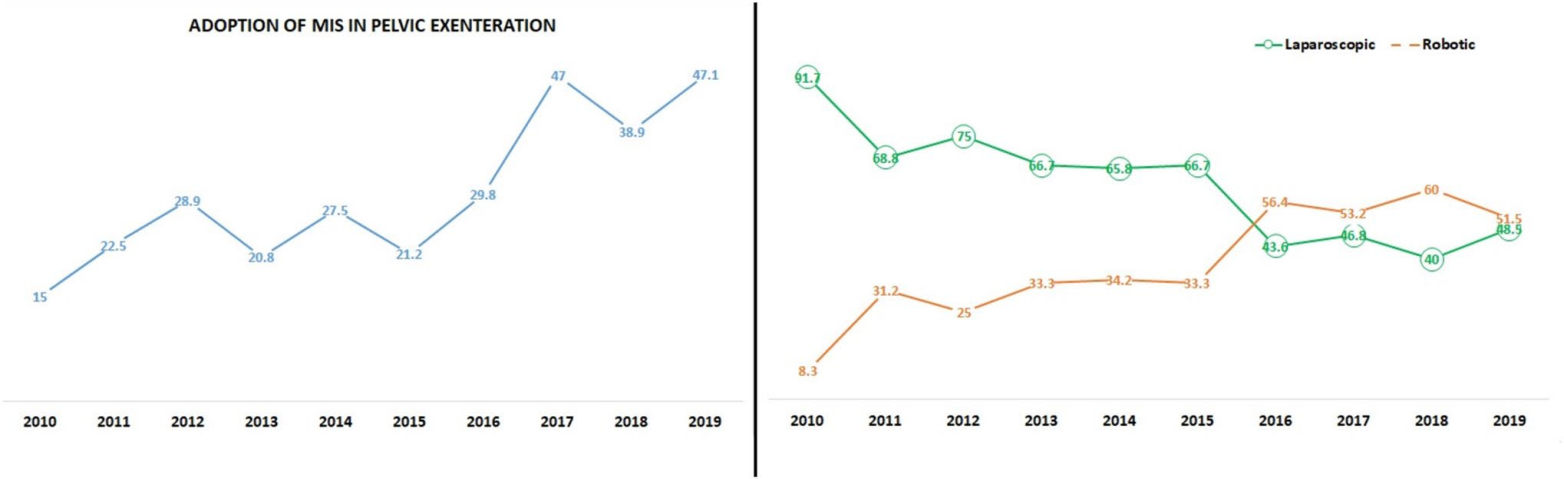
Trends in the use of minimally invasive pelvic exenteration over time

PE was performed by laparotomy in 705 (69.8%) patients and a minimally invasive approach in 304 (30.2%) patients (171 laparoscopic and 133 robotic-assisted procedures). Figure 2 illustrates the increased use of MIS in PE over time from 15% in 2010 to 47.1% in 2019. Robotic PE accounted for 8% of all minimally invasive PE in 2010 and the rate increased over time to 51.5% in 2019.

Patients who underwent minimally invasive PE were more often female (56.9% vs. 50.5%) and white (84.5% vs. 80.2%), had private insurance (51.6% vs. 45.5%), or medicare insurance (30.9% vs. 29.2%), underwent surgery in comprehensive community cancer programs (42.1% vs. 35.5%), and less often had T3–4 cancers (87.8% vs. 92.8%), and mucinous carcinomas (6.9% vs. 9.9%) (Table 1). Otherwise, patients in the two groups were balanced in terms of age, Charlson comorbidity index score, N stage, and neoadjuvant treatments.

**Table 1:** Baseline characteristics of patients in the original cohort

Factor	Group	Open (*n* = 705)	MIS (*n* = 304)	SMD
Mean age in years (SD)		58.1 (12.3)	57.9 (13.1)	0.014
Sex (%)	Male	349 (49.5)	131 (43.1)	0.129
	Female	356 (50.5)	173 (56.9)	
Race (%)	White	560 (80.2)	257 (84.5)	0.147
	Black	82 (11.7)	32 (10.5)	
	Asian	33 (4.7)	10 (3.3)	
	American Indian	6 (0.9)	2 (0.7)	
	Other	17 (2.4)	3 (1.0)	
Charlson Score (%)	0	576 (81.7)	247 (81.2)	0.057
	1	92 (13.0)	38 (12.5)	
	2	26 (3.7)	12 (3.9)	
	3	11 (1.6)	7 (2.3)	
Insurance status (%)	Medicaid	102 (14.9)	28 (9.2)	0.218
	Medicare	200 (29.2)	94 (30.9)	
	Other government	12 (1.8)	7 (2.3)	
	Private	312 (45.5)	157 (51.6)	
	Not insured	59 (8.6)	18 (5.9)	
Facility type (%)	Academic/Research Program	218 (31.3)	85 (28.1)	0.14
	Community Cancer Program	70 (10.1)	30 (9.9)	
	Comprehensive Community Cancer Program	247 (35.5)	127 (42.1)	
	Integrated Network Cancer Program	161 (23.1)	60 (19.9)	
Clinical T stage (%)	T1-2	47 (7.2)	35 (12.2)	0.172
	T3-4	609 (92.8)	251 (87.8)	
Clinical N stage (%)	N0	74 (10.9)	28 (9.6)	0.043
	N1-2	607 (89.1)	265 (90.4)	
Histology (%)	Adenocarcinoma	625 (88.7)	277 (91.1)	0.116
	Mucinous carcinoma	70 (9.9)	21 (6.9)	
	Signet ring cell carcinoma	10 (1.4)	6 (2.0)	
Grade (%)	Well-differentiated	31 (6.0)	18 (8.6)	0.113
	Moderately differentiated	395 (75.8)	153 (72.9)	
	Poorly differentiated	82 (15.7)	35 (16.7)	
	Undifferentiated	13 (2.5)	4 (1.9)	
Neoadjuvant systemic treatment (%)	No	102 (14.5)	39 (12.8)	0.048
	Yes	602 (85.5)	265 (87.2)	
Neoadjuvant radiation (%)	No	114 (16.4)	50 (16.8)	0.01
	Yes	581 (83.6)	248 (83.2)	

*MIS* minimally invasive surgery, *SMD* standard mean difference

Factors independently associated with an increased likelihood of having minimally invasive PE were Medicare insurance (OR: 1.77, 95% CI 1.06, 2.94, *p* = 0.028) and private insurance (OR: 1.8, 95% CI 1.11, 2.91, *p* = 0.016), whereas clinical T3–4 cancers were associated with a lower likelihood of having minimally invasive PE (OR: 0.59, 95% CI 0.37, 0.94, *p* = 0.026).

### Matching

After matching for sex, race, insurance type, facility type, T stage, tumor histology, and grade, 169 in the minimally invasive surgery group were matched to 338 patients in the open surgery group. The two groups were balanced regarding all baseline characteristics (Table 2).

**Table 2:** Baseline characteristics of patients in the matched cohort

Factor	Group	Total (*n* = 507)	Open (*n* = 338)	MIS (*n* = 169)	SMD
Mean age in years (SD)		57.4 (12.5)	57.6 (11.7)	57.1 (13.9)	0.039
Sex (%)	Male	232 (45.8)	154 (45.6)	78 (46.2)	0.012
	Female	275 (54.2)	184 (54.4)	91 (53.8)	
Race (%)	White	425 (83.8)	282 (83.4)	143 (84.6)	0.049
	Black	52 (10.3)	36 (10.7)	16 (9.5)	
	Asian	20 (3.9)	13 (3.8)	7 (4.1)	
	American Indian	3 (0.6)	2 (0.6)	1 (0.6)	
	Other	7 (1.4)	5 (1.5)	2 (1.2)	
Charlson score (%)	0	421 (83.0)	280 (82.8)	141 (83.4)	0.089
	1	66 (13.0)	45 (13.3)	21 (12.4)	
	2	14 (2.8)	10 (3.0)	4 (2.4)	
	3	6 (1.2)	3 (0.9)	3 (1.8)	
Insurance status (%)	Medicaid	53 (10.5)	35 (10.4)	18 (10.7)	0.1
	Medicare	137 (27.0)	88 (26.0)	49 (29.0)	
	Other government	5 (1.0)	4 (1.2)	1 (0.6)	
	Private	276 (54.4)	185 (54.7)	91 (53.8)	
	Not insured	36 (7.1)	26 (7.7)	10 (5.9)	
Facility type (%)	Academic/Research Program	147 (29.0)	98 (29.0)	49 (29.0)	0.078
	Community Cancer Program	53 (10.5)	33 (9.8)	20 (11.8)	
	Comprehensive Community Cancer Program	198 (39.1)	132 (39.1)	66 (39.1)	
	Integrated Network Cancer Program	109 (21.5)	75 (22.2)	34 (20.1)	
Clinical T stage (%)	T1–2	38 (7.7)	25 (7.6)	13 (8.1)	0.019
	T3–4	454 (92.3)	306 (92.4)	148 (91.9)	
Clinical N stage (%)	N0	58 (11.6)	40 (11.9)	18 (10.8)	0.033
	N1–2	444 (88.4)	296 (88.1)	148 (89.2)	
Histology (%)	Adenocarcinoma	458 (90.3)	308 (91.1)	150 (88.8)	0.1
	Mucinous carcinoma	41 (8.1)	26 (7.7)	15 (8.9)	
	Signet ring cell carcinoma	8 (1.6)	4 (1.2)	4 (2.4)	
Grade (%)	Well-differentiated	32 (6.3)	21 (6.2)	11 (6.5)	0.021
	Moderately differentiated	378 (74.6)	253 (74.9)	125 (74.0)	
	Poorly differentiated	85 (16.8)	56 (16.6)	29 (17.2)	
	Undifferentiated	12 (2.4)	8 (2.4)	4 (2.4)	
Neoadjuvant systemic treatment (%)	No	62 (12.2)	44 (13.0)	18 (10.7)	0.073
	Yes	445 (87.8)	294 (87.0)	151 (89.3)	
Neoadjuvant radiation (%)	No	70 (14)	45 (13.5)	25 (15.1)	0.045
	Yes	430 (86)	289 (86.5)	141 (84.9)	

*MIS* minimally invasive surgery, *SMD* standard mean difference, *SD* standard deviation

The matched cohort included 507 patients (54.2% female) with a mean age of 57.4 ± 12.5 years. Most patients were white (83.8%), had a Charlson score < 1 (83%), had private insurance (54.4%), had T3–4 cancers (92.3%) and N1–2 cancers (88.4%). Most tumors were moderately differentiated adenocarcinomas (74.6%), whereas mucinous and signet-ring cell carcinomas accounted for 9.7% of rectal cancers. Most patients in both groups received neoadjuvant systemic therapy (89.3% vs. 87%) and radiation therapy (84.9% vs. 86.5%). Conversion from MIS to open surgery was undertaken in 16.6% of patients (Table 2). The median follow-up in the matched cohort was 48.4 (IQR: 28.4, 76.2) months.

### Outcomes

The matched groups had similar short-term outcomes, except for a shorter hospital stay in favor of minimally invasive PE (median: 6 vs. 8 days, *p* < 0.001). Minimally invasive PE was associated with a non-significant decrease in the odds of 30-day mortality (OR: 0.33, 95% CI 0.04, 2.75, *p* = 0.306), 90-day mortality (OR: 0.29, 95% CI 0.07, 1.33, *p* = 0.113), and 30-day unplanned readmissions (OR: 0.96, 95% CI 0.47, 1.95, *p* = 0.904) (Table 3). Missing data were < 10% for all outcomes. The lack of statistical significance for short-term outcomes was confirmed in the Mantel–Haenszel sensitivity analysis, which showed that the p values of 30- and 90-day mortality and readmission remained insignificant at different sensitivity levels, indicating robust outcomes that were not sensitive to change caused by unobserved confounders (Supplementary Table 2).

**Table 3:** Matched outcomes of open and minimally invasive pelvic exenteration

Factor	Group	Open (n = 338)	MIS (*n* = 169)	*p* value
Median hospital stay in days [IQR]		8 [6, 11.25]	6 [4, 8.75]	** < 0.001**
30-day mortality (%)	No	332 (98.2)	168 (99.4)	0.433
	Yes	6 (1.8)	1 (0.6)	
90-day mortality (%)	No	323 (96.1)	167 (98.8)	0.104
	Yes	13 (3.9)	2 (1.2)	
30-day readmission (%)	No readmission	306 (91.1)	154 (91.7)	0.608
	Planned readmission	5 (1.5)	2 (1.2)	
	Unplanned readmission	25 (7.4)	12 (7.1)	
Surgical margins (%)	Negative	284 (85.0)	145 (87.3)	0.586
	Positive	50 (15.0)	21 (12.7)	
Median number of examined lymph nodes [IQR]		15 [11, 22]	15 [12, 21]	0.84
Lymph node yield < 12 (%)	No	250 (74.0)	136 (81.0)	0.096
	Yes	88 (26.0)	32 (19.0)	
Overall survival (%)	Alive	193 (57.1)	112 (66.3)	0.054
	Dead	145 (42.9)	57 (33.7)	
Median follow-up in months [IQR]		50.9 [28.3, 76.6]	44.8 [28.5, 73.6]	0.305
Median number of positive lymph nodes [IQR]		0 [0, 2]	0 [0, 2]	0.586

Bold text in p value column indicates statistical significance

*MIS* minimally invasive surgery, *IQR* interquartile range

**Fig. 3:**
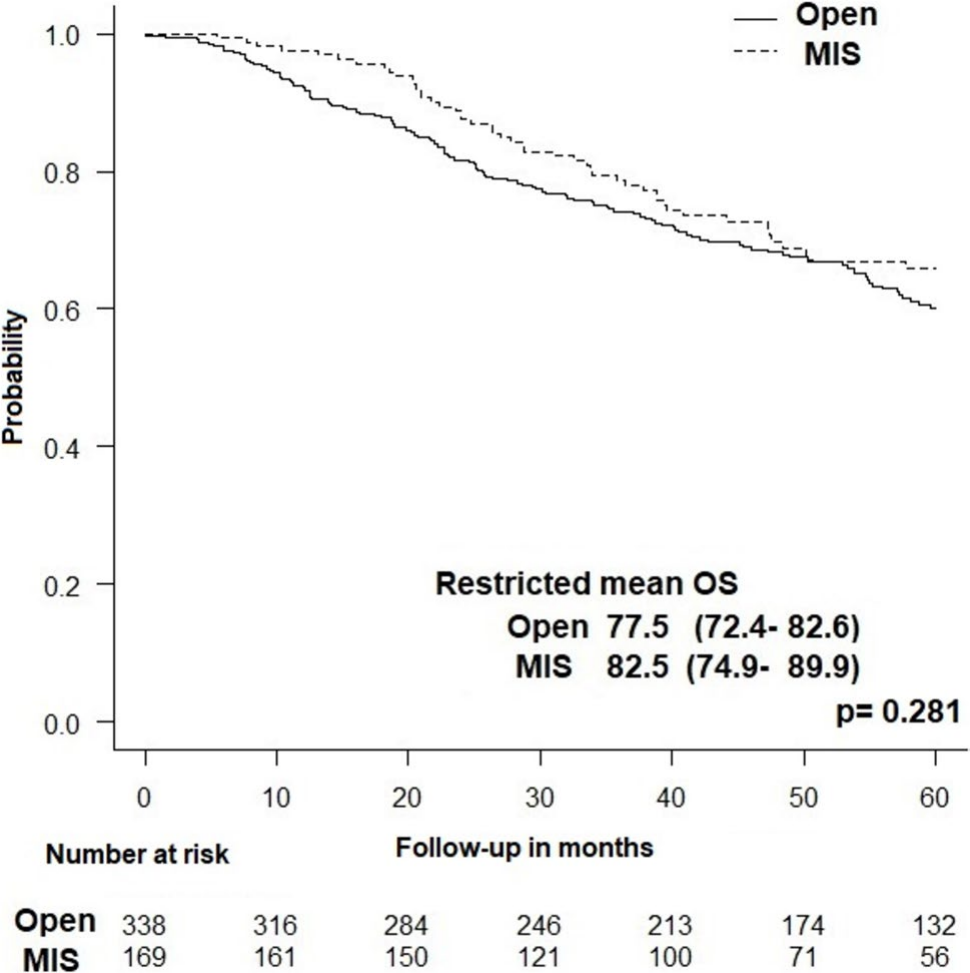
Kaplan–Meier curve showing the difference in overall survival between minimally invasive and open pelvic exenteration

Pathologic outcomes were also similar among the two groups as minimally invasive and open PE was associated with similar rates of positive surgical margins (12.7% vs. 15%, *p* = 0.586) and suboptimal (< 12) lymph node yield (19% vs. 26%, *p* = 0.096). The 5-year OS was slightly higher in the minimally invasive group with a borderline significance (66.3% vs. 57.1%, *p* = 0.054). Similarly, the Kaplan–Meier test showed that minimally invasive PE had longer restricted mean OS than open surgery (82.5 vs. 77.5 months), yet without reaching statistical significance (*p* = 0.281) **(**Fig. 3).

### Laparoscopic versus robotic-assisted PE

Comparing 103 laparoscopic and 66 robotic-assisted PE in the matched cohort showed that robotic-assisted PE was associated with significantly lower odds of conversion to open surgery (OR: 0.15, 95% CI 0.04–0.51, *p* = 0.003) and shorter hospital stay (median: 5 vs. 7 days, *p* = 0.026) than did laparoscopic PE. The two groups had similar rates of 30-day mortality, 90-day mortality, 30-day readmissions, and positive surgical margins. The 5-year OS was also similar between laparoscopic and robotic-assisted PE (66% vs. 66.7%, *p* = 0.931) (Table 4).

**Table 4:** Outcomes of laparoscopic and robotic-assisted pelvic exenteration

Factor	Group	Laparoscopic (*n* = 103)	Robotic (*n* = 66)	*p* value
Conversion (%)	No	78 (75.7)	63 (95.5)	**0.001**
	Yes	25 (24.3)	3 (4.5)	
Median hospital stay in days [IQR]		7 [5, 9]	5 [4, 7]	**0.026**
30-day mortality (%)	No	103 (100.0)	65 (98.5)	0.391
	Yes	0 (0.0)	1 (1.5)	
90-day mortality (%)	No	103 (100.0)	64 (97.0)	0.151
	Yes	0 (0.0)	2 (3.0)	
30-day readmission (%)	No readmission	95 (92.2)	59 (90.7)	0.36
	Planned readmission	2 (1.9)	0 (0.0)	
	Unplanned readmission	6 (5.9)	6 (9.3)	
Surgical margins (%)	Negative	88 (87.1)	57 (87.7)	0.858
	Positive	13 (12.9)	8 (12.3)	
Median number of examined lymph nodes [IQR]		15 [11, 22]	15 [12, 21]	0.84
Overall survival (%)	Alive	68 (66.0)	44 (66.7)	0.931
	Dead	35 (34.0)	22 (33.3)	

Bold text in *p* value column indicates statistical significance

*IQR* interquartile range

## Discussion

The present study found that open and minimally invasive PE had similar short-term mortality, readmission, and surgical quality parameters. The main benefit of MIS was a two-day shorter hospital stay and potentially better OS than open PE. Robotic-assisted PE was associated with less conversion to open surgery and shorter hospital stay than did laparoscopic PE, otherwise 30-day mortality, 90-day mortality, readmissions, positive margins, and survival were similar between laparoscopic and robotic-assisted PE.

PE accounted for approximately 2% of procedures performed for stage III rectal cancer, which is expected because such multi-visceral extensive resection is preserved for select cases with infiltration of neighboring structures. Approximately 70% of PE was performed by laparotomy, consistent with the overall preference for the open approach to PE for rectal cancer and other pelvic malignancies [8, 9, 13]. Patients who underwent minimally invasive PE were more often women, perhaps because it was assumed that using a minimally invasive approach to multi-visceral resection would be more technically challenging in men given their known anatomic limitations, including narrow pelvises [14]. Also, having medicare or private insurance was associated with a 77–80% higher chance of undergoing minimally invasive PE. On the other hand, patients with more aggressive rectal cancer pathologies, including mucinous carcinomas, were more likely to undergo open PE as more challenging resections may be expected with mucinous neoplasms [15]. Although PE is mainly indicated for rectal cancers infiltrating neighboring structures, approximately 8% of patients in the matched cohort had clinical T1–2 tumors. These patients may have had coexisting uterine lesions that required hysterectomy in the setting of proctectomy and thus were considered to have PE.

Minimally invasive PE might be associated with better recovery than open PE as implied by a 2-day shorter hospital stay. This benefit is probably attributable to the nature of MIS which entails smaller incisions and less post-operative pain [16]. This finding was different from the findings of two previous studies [7, 8] that reported similar hospital stays between open and minimally invasive PE. However, since the previous studies did not match open and MIS for baseline confounders, selection bias may have contributed to the lack of significant difference in stay.

The odds of short-term mortality were similar between minimally invasive and open PE. 30-day mortality is usually related to immediate post-operative adverse events though it can be a direct result of pre-existing comorbidities [17]. Previous comparative studies [7, 8] found similar post-operative morbidity after open and minimally invasive PE. Conversely, a population-based study found that using a minimally invasive approach to colorectal resections conferred a 21% reduction in 30-day mortality compared to open resections [18]. Our results were consistent with former findings of no significant differences either because of the small numbers included or because the morbidity associated with PE is too extensive to be mitigated by just using a minimally invasive approach [6]. The 30-day mortality rate in our cohort was 0.6% after minimally invasive PE and 1.8% after open PE, and both were close to the median perioperative mortality rate (2%) reported in a systematic review [19]. However, the mortality rates doubled at 90 days to 1.2% and 3.9% after minimally invasive and open PE, respectively. This striking increase in mortality at 90 days is not unexpected as 90-day mortality captures mortality from multiple causes, most of which are related to the general and functional status of the patients, rather than to surgery itself [20].

Some surgeons may have concerns about using MIS in major resections such as PE. Their main concerns pertain to whether minimally PE could provide equivalent pathologic and oncologic outcomes to the traditional open approach. Our analysis showed that minimally invasive PE conferred adequate surgical resections as shown by an R0 rate of 87.3%, slightly higher than that of open PE. The rate of R0 resection in both groups was higher than the rate of 74% reported in previous systematic reviews [6, 21], yet close to the rate of 88.5% reported in a cohort study [8]. Furthermore, the rate of suboptimal lymph node yield was similar between minimally invasive and open PE. These findings indicated that minimally invasive PE might be oncologically adequate for LARC as it achieved comparable resection quality to the open approach. Minimally invasive PE might be associated with a survival benefit over open PE as showed by a borderline significantly higher OS rate and longer mean survival. However, this potential survival benefit was not reproduced in other studies as Kazi et al. [8] reported no differences in 3-year OS or recurrence-free survival between the two approaches. Similarly, open and minimally invasive PE for gynecologic cancers were reported to have similar disease-free survival and cancer-specific survival [13].

The two MIS approaches used in the study had similar clinical, pathologic, and survival outcomes. However, robotic-assisted surgery was associated with less conversion to open surgery and shorter stays than laparoscopic PE, consistent with the previously reported benefits of robotic-assisted surgery for advanced rectal cancer [22]. Therefore, the robotic platform may provide an option to perform a minimally invasive PE in patients deemed at high risk for conversion to open surgery.

The present study provided a detailed analysis of the short-term and survival outcomes of minimally invasive compared to open PE. The use of a large national database and the application of matching techniques served to reduce selection bias in terms of observable confounders. The present study represents one of the largest analyses that compared different surgical approaches to PE. However, some limitations to the study should be acknowledged. An important limitation is the retrospective nature of the study and the inherent limitations of using databases in research, such as missing data, misclassifications, and lack of standard criteria for the selection of patients for each group. As with all retrospective data analyses, the risk of selection bias will remain despite the efforts to minimize it using matching techniques. While open and minimally invasive PE were matched for some important confounders that may impact the outcomes, other factors, such as body mass index, comorbidity profile, and type of PE, were not accounted for in the analysis. However, the sensitivity analysis confirmed that the primary results were not sensitive to change caused by unobserved confounders. It should be noted that PE includes a diverse group of operations, and thus it is likely that the minimally invasive approach was selected for a suitable cohort of patients for this procedure, in whom no bony structures were infiltrated by cancer. We could not account for the type of PE, whether standard or extended, in our matching and thus the similar R0 rates between open and minimally invasive PE may have been confounded by different types of exenterations. However, PE involving bone resection accounts for less than 10% of procedures and thus may not have imposed significant selection bias [23]. Moreover, some relevant outcomes, such as operative time and blood loss, were not assessed because they were not reported in the database used. The type of PE was not reported and there might have been variations in the codes used to record PE in each participating hospital. Data on the technical skills needed to master minimally invasive PE and access to the required resources were not available. Thus, the outcomes of minimally invasive PE may be attributed to highly skilled MIS surgeons. Implementation of the standards of Enhanced Recovery After Surgery (ERAS) may have also affected the short-term outcomes of both approaches, however, there was no information about ERAS in the database used. Moreover, the timing of the procedures as elective, urgent, and emergent was not reported in the NCDB, and it is possible that open PE was more frequently used in emergency cases. It is important to note the possibility of incomplete matching as the matched sample size was < 50% of the original cohort which may have a potential influence on the outcomes. Lastly, the database does not include a homogenous definition of conversion and different surgeons may have employed different definitions. Therefore, laparoscopic and robotic surgical differences could have potentially been attributed to differences in definitions of conversion.

## Conclusions

Minimally invasive PE provided similar pathologic and survival outcomes to open PE with comparable rates of short-term mortality and significantly shorter hospital stays. Robotic-assisted PE was associated with a lower likelihood of conversion to open surgery and shorter hospital stays than laparoscopy. This finding should be cautiously interpreted because of  the risk of selection of bias due to the lack of a homogenous definition of conversion to open surgery.

## Supplementary Information

Below is the link to the electronic supplementary material.

Supplementary file1 (DOCX 19 KB)

Supplementary file2 (DOCX 14 KB)
