# Supplementary material for: Propensity-score matched outcomes of minimally invasive and open pelvic exenteration in locally advanced rectal cancer
Source: Updates Surg. 2025 Jan 16;77(2):267–76. doi: 10.1007/s13304-025-02102-7 (PMC11961522; doi:10.1007/s13304-025-02102-7)
Supplement: Supplementary file 2 — Supplementary file2 (DOCX 14 KB) [file 13304_2025_2102_MOESM2_ESM.docx]

**Supplementary table 2.** Results of sensitivity analyses

| **Gamma** | **Lower P-Value** | **Upper P-Value** |
| --- | --- | --- |
| **30-day mortality** | | |
| 0.5 | 0.720 | 0.590 |
| 1 | 0.077 | 0.599 |
| 1.5 | 0.985 | 0.923 |
| 2 | 0.809 | 0.052 |
| **90-day mortality** | | |
| 0.5 | 0.250 | 0.273 |
| 1 | 0.883 | 0.634 |
| 1.5 | 0.319 | 0.531 |
| 2 | 0.403 | 0.055 |
| **30-day readmission** | | |
| 0.5 | 0.925 | 0.980 |
| 1 | 0.888 | 0.436 |
| 1.5 | 0.657 | 0.419 |
| 2 | 0.592 | 0.472 |
